# Imprisonment following discharge from mental health units: A developing trend in New Zealand

**DOI:** 10.3389/fpsyt.2023.1038803

**Published:** 2023-01-26

**Authors:** Jeremy Skipworth, Nick Garrett, Krishna Pillai, Rees Tapsell, Brian McKenna

**Affiliations:** ^1^Auckland Regional Forensic Psychiatry Services, Waitemata District Health Board, Auckland, New Zealand; ^2^Faculty of Medical and Health Sciences, University of Auckland, Auckland, New Zealand; ^3^Faculty of Health and Environmental Sciences, Auckland University of Technology, Auckland, New Zealand; ^4^Mental Health and Addictions Service, Waikato District Health Board, Hamilton, New Zealand; ^5^Centre for Forensic Behavioural Science, Swinburne University of Technology, Melbourne, VIC, Australia

**Keywords:** prison, mental health, inpatient, violence, coercion

## Abstract

**Introduction:**

Contemporary models of care for people with mental disorders continue to shift to community-based care, requiring fewer inpatient mental health beds, shorter inpatient lengths of stay, and less use of coercion. It has been suggested that some mentally unwell people, whose behavior can no longer be safely contained in overstretched mental health units where seclusion and restraint are discouraged, are now left to the criminal justice system to manage. It is unclear whether the risk of imprisonment following discharge from a mental health unit has increased over recent years.

**Methods:**

A quantitative, retrospective cohort study design was used to investigate any association between an acute inpatient mental health service admission in Aotearoa (New Zealand), and referral to a prison mental health team within 28 days of hospital discharge, from 2012 to 2020. Data were extracted from the national mental health dataset managed by the Ministry of Health.

**Results:**

Risk of imprisonment within 28 days of inpatient discharge increased over the study period. People experiencing this outcome were more likely to be younger, male, of Mâori or Pacific ethnicity, presenting with substance use and psychotic disorders who were aggressive or overactive, and were subject to coercive interventions such as seclusion and compulsory treatment during their admission.

**Discussion:**

We concluded that contemporary models of less coercive predominantly community based mental health care may be increasingly reliant on the criminal justice system to manage aggressive and violent behavior driven by mental illness. It is argued from a human rights perspective that mental health inpatient units should retain the capacity to safely manage this type of clinical presentation.

## Introduction

Deinstitutionalisation, or the process of shifting from institutionally based mental healthcare to community-based care has been happening around the world since the 1950s. It is now well established that with sufficient resourcing of community-based services, most people who were previously in long-term inpatient psychiatric care can be successfully treated in the community (1), where there is evidence for a higher quality of life than formerly experienced in institutional care (2). However, for a small number of people with mental disorders, hospital admission is necessary to provide safe and appropriate care (3). More controversially, the number of inpatient psychiatric beds needed to ensure appropriate care for the population is the subject of ongoing debate. The Treatment Advocacy Center have advocated for a bed target of approximately 50 beds per 100,000 population (4).

In Aotearoa (New Zealand), the process of deinstitutionalisation saw the number of residents in inpatient mental health facilities fall from 350 per 100,000 population in the 1970s to about 50 per 100,000 population by the late 1990s (5). Over the last two decades psychiatric bed numbers have continued to fall and now average 28 beds per 100,000 population (Welsh, 2022^1^). At the same time, the average length of stay (LOS) has fallen to 18 days, while the occupancy of many inpatient services often exceeds 100% (Memorandum from the Ministry of Health to the Minister of Health, March, 2021).

Internationally, Aotearoa is not unusual in dramatically reducing mental health bed numbers, while at the same time reducing LOS. In Australia, in 2019–2020 the national rate of public sector mental health beds per 100,000 population had fallen to 27.5 (6). In the United Kingdom, Tyrer et al. (7) raised concerns that the number of mental health beds had fallen from 100 beds per 100,000 population in the late 1990s to less than 50 per 100,000 population by 2014, with the average LOS reducing to 15 days. In the United States, mental health beds were reduced to 25 beds per 100,000 population, with an average LOS of only 6 days (3). The same trends have also been observed in Central and Eastern Europe (8).

Further complicating matters, in Aotearoa as elsewhere, a paradigm shift toward a human rights-based framework has challenged more traditional models of compulsory inpatient care, as has our increasing awareness of the negative impact of trauma and coercive models of compulsory care, seclusion, and restraint (9). Government policy now encourages the elimination of seclusion and restraint. Service providers are also encouraged to reduce the application of compulsory treatment in the community to Mâori (the indigenous people of Aotearoa), who are subject to more compulsory treatment than are non-Mâori (10). This is despite evidence that Mâori suffer higher rates of serious mental illness (11, 12). Additionally, new facilities are now being constructed to embrace less coercive models of care, staff training has an emphasis on preventing the use of restrictive practices (13) and legislative reform of the Mental Health Act is proposed to move away from coercive practices, and toward a capacity-based framework for compulsory care (14).

However, emerging from the laudable motives to reduce iatrogenic harm caused by coercive care, concerns are now raised that with shortages of available acute mental health beds and new models of less coercive inpatient care, those who cannot easily be cared for in non-coercive environments are at risk of being progressively denied access to a critical part of the continuum of care needed for this service user population.

A primary concern in this regard, is the suggestion that this shift may lead to increased criminalization of behavior driven by mental illness, and in some cases to custodial remand as an alternative to inpatient treatment (2, 8, 15). The prison remand population in Aotearoa increased from 1,800 in 2012, to 3,409 in 2020 (16). This increase in the remand prison population was in contrast to the number of individuals charged with a criminal offense, which almost halved following a peak in 2009/2010 of more than 120,000 individuals to 67,123 in 2020/2021 (17).

It is against this background of New Zealand’s mental health bed reduction below international benchmark standards and coercive care being increasingly discouraged that we sought to investigate whether there was any evidence of a trend toward increasing custodial remand for persons with serious mental illness recently discharged from inpatient mental health units. Anecdotally, the authors were aware of many cases of psychiatric inpatients arrested after an assault in the inpatient unit and remanded to prison, and other remand prisoners recently discharged from a psychiatric inpatient unit but still subject to the Mental Health Act.

We therefore undertook a secondary analysis of available operational data. The study period was limited to recent years from 2012 to 2020 (when nationally collected data was sufficiently reliable to interrogate). If a trend toward increasing numbers of recently discharged remand prisoners was revealed, we were interested to better understand whether this trend could be explained by changes in inpatient characteristics, as well as broader social factors which may be contributing to the observed trend, such as the availability of supported accommodation and illicit drugs.

## Materials and methods

A quantitative, retrospective cohort study design was used to determine any association between the exposure event (an acute inpatient mental health service admission) and the outcome event (opening a referral to a prison mental health team within 28 days of hospital discharge), over time. Data was extracted for all discharges from an acute mental health inpatient unit in New Zealand over a 9-year period, from 1 January 2012 to 31 December 2020.

The outcome event was not linked to Justice data on custodial remands. However, universal screening for mental health contact occurred throughout the study period on the day of reception to prison, utilizing a screening tool which combines the Brief Jail Mental Health Screen and the English Mental Health Screen (18). This should result in all people recently discharged from a mental health unit being referred to a prison mental health team. The authors’ experience is that this screening is very effective in identifying recently discharged patients. We concluded there would be very few people remanded to prison within 28 days of discharge from a mental health unit who were not captured in the dataset.

All data were extracted from the Programme for the Integration of Mental Health Data (PRIMHD). This national data set of mental health and addiction sector activity is managed by Te Pou, on behalf of the Ministry of Health (19). The data was initially extracted and downloaded by the data manager at Te Pou and released to the researchers as an anonymous Excel spreadsheet, which was then imported into SAS version 9.4^2^ for data cleaning and analysis.

### Measures

The data extract included demographic, clinical, social, and model of care variables to describe the characteristics of the cohort, and determine the influence of potential confounding variables on the outcome variable (reception to remand prison).

*Demographic information* included gender, ethnicity, and age.

*Clinical information* included clinical diagnosis and individual Health of the Nation Outcome Scale (HoNOS) scores (20) at admission and discharge. Clinical diagnoses were determined from International Classification of Diseases (ICD) codes reported at discharge from mental health services. The ICD codes were collapsed into groupings based on numbers and relevance. These groupings were mental and behavioral disorders due to psychoactive substance use (F1, 6.1%), schizophrenia, schizotypal, delusional, and other non-mood psychotic disorders (F2, 25.7%), mood (affective) disorders (F3, 21.9%), anxiety, dissociative, stress-related, somatoform and other non-psychotic mental disorders (F4, 6.8%), other mental health diagnoses (F0 2.4, F5 0.5, F6 4.4, F7 0.1, F8 0.4, and F9 0.4%), and other diagnoses which included: diseases of the nervous system (G 0.6%), symptoms and signs involving cognition, perception, emotional state and behavior (R4, 0.6%), general symptoms and signs–illness unspecified (R69, 0.2%), factors influencing health status, and contact with health services (Z, 5.3%). In a minority of cases there was no clinical diagnosis at discharge, which usually records an admission for observation for a suspected condition ruled out at discharge.

The HoNOS scores were categorized into quartiles and because many individuals did not have HoNOS scores reported, a separate category was created for those with no HoNOS data, recognizing that lack of HoNOS scores may indicate specific operational conditions.

*Social information* was derived from the HoNOS, which considers a range of social variables including relationships, daily living, living conditions, and occupation/activities (HoNOS items 9–12).

*Model of care* information included LOS, whether seclusion was used during the admission, legal status at discharge (whether subject to Mental Health Act order or not), and the geographical region of the inpatient admission, to see whether different model of care approaches in different regions were yielding different results.

### Analysis

An initial descriptive analysis of the cohort variables was undertaken, using sums, and percentages. A Cochran–Armitage trend test examined the observed trend in the rate of reception to remand prison by discharge year. Inferential analyses were then untaken using repeated measures logistic regression with the outcome variable of referral to a prison mental health team within 28 days of inpatient unit discharge, the repeated measures model utilized a compound symmetry covariance structure for repeated individual effects. Firstly, bivariate associations were examined between the outcome variable and each of the other variables individually. Variables with a *p*-value of 0.2 or less were then considered for inclusion in the multiple variable model building process, from which the best subset of significant variables determined the final multiple variable models.

### Ethics

Given that this was observational research, a full ethics approval was not required by the Ministry of Health’s Health and Disability Ethics Committees. A letter of approval was obtained as a result of an expedited research application.

## Results

### Cohort description

The national dataset identified 95,206 inpatient admissions over the 9 year study period, involving 46,299 individuals, some of whom were admitted on more than one occasion (range 1–84, mean 2.1).

A total of 708 of the 95,206 hospital admissions (0.7%) were followed by a mental health team contact in prison within 28 days of inpatient discharge. This involved 575 individuals, some of whom had been admitted on more than one occasion in the study period. Among this group, the number of inpatient events per individual ranged from 1 to 6, averaging 1.2.

Despite reasonably even gender split of hospital admissions (51.4% male), subsequent mental health contact in prison was disproportionately male (83.5%). In terms of ethnicity, whereas Mâori were 29.8% of hospital admissions, they accounted for 51.7% of subsequent prison referrals. Younger age groups were also more heavily represented in the outcome events. For example, 24.8% of inpatient admissions were under age 25, whereas 33.3% prison referrals were under age 25 (for detail see Table 1).

**TABLE 1:** Demographics of mental health inpatient discharge events (2012–2020).

		All inpatient events		Prison follow-up events at 28 days		Rate
		No	%	No	%	%
**Socio-demographic variables**						
Gender	Female	46,314	48.6	117	16.5	0.3
	Male	48,892	51.4	591	83.5	1.2
Ethnicity	Mâori	28,340	29.8	364	51.4	1.3
	Pacific	5,372	5.6	55	7.8	1.0
	Other	6,036	6.3	34	4.8	0.6
	NZ European/Pakeha	55,458	58.3	255	36.0	0.5
Age	16–20	10,643	11.2	81	11.4	0.8
	20–25	12,988	13.6	155	21.9	1.2
	26–30	11,149	11.7	131	18.5	1.2
	31–40	17,700	18.6	189	26.7	1.1
	41–50	17,145	18.0	94	13.3	0.5
	51+	25,581	26.9	58	8.2	0.2
**Clinical characteristics**						
Year discharged	2012	9,985	10.5	56	7.9	0.6
	2013	10,581	11.1	59	8.3	0.6
	2014	10,912	11.5	77	10.9	0.7
	2015	10,694	11.2	84	11.9	0.8
	2016	11,015	11.6	92	13.0	0.8
	2017	10,645	11.2	85	12.0	0.8
	2018	10,502	11.0	80	11.3	0.8
	2019	10,519	11.0	98	13.8	0.9
	2020	10,353	10.9	77	10.9	0.7
Primary diagnosis [International Classification of Diseases (ICD) 10 codes]	Psychoactive substance use (F1)	5,983	6.3	108	15.3	1.8
	Psychotic disorders (F2)	24,967	26.2	224	31.6	0.9
	Mood disorders (F3)	20,873	21.9	74	10.5	0.4
	Anxiety and other non-psychotic mental disorders (F4)	6,040	6.3	41	5.8	0.7
	Other mental health diagnoses (F)	5,607	5.9	57	8.1	1.0
	Other diagnoses	8,037	8.4	47	6.6	0.6
	Not diagnosed	23,699	24.9	157	22.2	0.7
Total HoNOS–at admission	0–9	13,496	14.2	77	10.9	0.6
	10–13	15,511	16.3	90	12.7	0.6
	14–18	17,333	18.2	130	18.4	0.8
	19+	16,600	17.4	222	31.4	1.3
	Not done	32,266	33.9	189	26.7	0.6
Total HoNOS–at discharge	0–2	13,073	13.7	38	5.4	0.3
	3–4	11,678	12.3	54	7.6	0.5
	5–8	19,892	20.9	133	18.8	0.7
	9+	18,943	19.9	279	39.4	1.5
	Not done	31,620	33.2	204	28.8	0.6
**Model of care variables**						
Region	Auckland RFPS	28,794	30.2	168	23.7	0.6
	Canterbury RFPS	17,058	17.9	112	15.8	0.7
	Central RFPS	17,574	18.5	130	18.4	0.7
	Midland RFPS	22,664	23.8	221	31.2	1.0
	Southern RFPS	9,116	9.6	77	10.9	0.8
Seclusion events	0	87,114	91.5	497	70.2	0.6
	1	5,489	5.8	127	17.9	2.3
	2+	2,603	2.7	84	11.9	3.2
Length of stay (days)	0–6	29,662	31.2	318	44.9	1.1
	7–12	20,718	21.8	150	21.2	0.7
	13–24	22,080	23.2	132	18.6	0.6
	25+	22,746	23.9	108	15.3	0.5
Mental Health Act at discharge	Yes	60,842	63.9	547	77.3	0.9
	No	34,364	36.1	161	22.7	0.5
**Total**		95,206		708		0.7

Nearly a third of the 708 admissions followed by mental health contact in prison occurred within 7 days of discharge (*n* = 229 of 708; 32.3%). Seclusion was used at more than three times the rate among discharged people remanded to prison (8.5% as compared to 29.8%). Of all inpatient events, 63.9% were discharged on a Mental Health Act order, while 77.3% remanded to prison were discharged under an order (see Table 1 for detail of clinical and model of care variables). Total HONOS score at hospital admission averaged 14.8, and at discharge averaged 6.7.

The proportion of inpatient discharges subsequently seen by mental health services in prison within 28 days of discharge increased during the study period, from 0.6% of discharges in 2012, to a high of 0.9% of discharges in 2019 (see Table 1). Examining this trend with the Cochran–Armitage trend test demonstrated that this was a statistically significant trend (*p* = 0.0025).

### Bivariate results

Results of the bivariate analyses demonstrated significant associations with the majority of variables and the outcome measure of mental health contact in prison.

Examination of the socio-demographic variables demonstrated significant differences by gender (*p* < 0.0001), ethnicity (*p* < 0.0001), and age group (*p* < 0.0001). Expressed as an odds ratio (OR), Mâori had an OR of 2.76 and Pasifika 2.19 times NZ European/Pakeha; and men had an OR of 5.32 times women, of custodial remand within 28 days discharge (see Table 2).

**TABLE 2:** Bivariate associations with custodial remand within 28 days: socio-demographic and clinical characteristics.

		No. of discharge events	% Prison follow-up at 28 days	OR	95% CI	*p*-value
**Socio-demographics variables**						
Gender	Female	46,314	0.26	1.00	–	<0.0001
	Male	48,892	1.27	5.32	4.18, 6.79	
Ethnicity	Mâori	28,340	1.33	2.76	2.29, 3.33	<0.0001
	Pacific	5,372	1.04	2.19	1.57, 3.07	
	Other	6,036	0.58	1.28	0.86, 1.90	
	NZ European/Pakeha	55,458	0.50	1.00	–	
Age	16–20	10,643	0.84	3.31	2.24, 4.87	<0.0001
	20–25	12,988	1.24	5.02	3.53, 7.13	
	26–30	11,149	1.24	5.26	3.70, 7.47	
	31–40	17,700	1.11	4.81	3.44, 6.73	
	41–50	17,145	0.58	2.58	1.77, 3.76	
	51+	25,581	0.24	1.00	–	
**Clinical characteristics**						
Year discharged	2012	9,985	0.59	1.00	–	0.03
	2013	10,581	0.61	1.01	0.66, 1.54	
	2014	10,912	0.77	1.35	0.88, 2.07	
	2015	10,694	0.80	1.44	0.95, 2.19	
	2016	11,015	0.86	1.51	1.00, 2.28	
	2017	10,645	0.81	1.52	1.02, 2.27	
	2018	10,502	0.77	1.44	0.96, 2.15	
	2019	10,519	0.99	1.80	1.22, 2.67	
	2020	10,353	0.82	1.50	1.00, 2.24	
Primary diagnosis (ICD 10 codes)	Psychoactive substance use (F1)	5,983	1.87	2.91	2.19, 3.88	<0.0001
	Psychotic disorders (F2)	24,967	0.94	1.37	1.07, 1.76	
	Mood disorders (F3)	20,873	0.40	0.57	0.42, 0.78	
	Anxiety and other non-psychotic mental disorders (F4)	6,040	0.73	1.17	0.81, 1.71	
	Other mental health diagnoses (F)	5,607	1.11	1.84	1.25, 2.72	
	Other diagnoses	8,037	0.60	0.96	0.66, 1.41	
	Not diagnosed	23,699	0.69	1.00	–	
**Model of care variables**						
Region	Auckland RFPS	28,794	0.60	1.00	–	0.0005
	Canterbury RFPS	17,058	0.72	1.13	0.85, 1.49	
	Central RFPS	17,574	0.78	1.27	0.97, 1.67	
	Midland RFPS	22,664	1.05	1.66	1.30, 2.11	
	Southern RFPS	9,116	0.84	1.54	1.12, 2.12	
Seclusion events	0	87,114	0.60	1.00	–	<0.0001
	1	5,489	2.42	3.51	2.76, 4.47	
	2+	2,603	3.30	4.47	3.36, 5.95	
Length of stay (days)	0–6	29,662	1.13	1.00	–	<0.0001
	7–12	20,718	0.77	0.58	0.46, 0.72	
	13–24	22,080	0.64	0.42	0.33, 0.55	
	25+	22,746	0.49	0.68	0.55, 0.84	
Mental Health Act at discharge	Yes	60,842	0.95	1.70	1.39, 2.07	<0.0001
	No	34,364	0.49	1.00	–	

Clinical measures demonstrated significant differences by year discharged (*p* = 0.03), primary diagnosis (*p* < 0.0001), (see Table 2) and many of the HoNOS items at both admission and discharge (see Table 3). The risk of prison referral within 28 days of discharge, expressed as an OR, was significantly higher for individuals with a diagnosis of a substance use disorder, or a psychotic disorder (OR = 2.91 and 1.37, respectively; *p* < 0.0001) (see Table 2). An inpatient discharge rating of substance abuse (HoNOS item 3) as severe to very severe also increased the risk of the outcome event (OR = 6.71, *p* < 0.0001) (see Table 3). In contrast, individuals with mood disorders were at reduced risk (OR = 0.57; *P* = < 0.0001) (see Table 2).

**TABLE 3:** Bivariate associations with custodial remand within 28 days: Health of the Nation Outcome Scale (HoNOS) admission and discharge item scores.

		No of discharge events	% Prison follow-up at 28 days	OR	95% CI	*p*-value
**HoNOS items at admission**						
1. Aggression/Overactivity	0. No problem	18,317	0.48	1.00	–	<0.0001
	1. Minor problem	16,224	0.60	1.15	0.86, 1.55	
	2. Mild problem	13,431	0.78	1.43	1.06, 1.92	
	3. Moderately severe problem	9,451	1.14	1.93	1.42, 2.64	
	4. Severe to very severe problem	5,409	2.14	3.57	2.60, 4.90	
	Not done	32,374	0.60	1.19	0.91, 1.55	
2. Self-harm	0. No problem	35,171	0.88	1.00	–	0.02
	1. Minor problem	7,105	0.82	0.94	0.68, 1.30	
	2. Mild problem	6,118	0.64	0.80	0.57, 1.14	
	3. Moderately severe problem	7,944	0.63	0.81	0.60, 1.10	
	4. Severe to very severe problem	6,360	0.91	1.24	0.94, 1.65	
	Not done	32,508	0.59	0.76	0.62, 0.93	
3. Substance abuse	0. No problem	28,407	0.37	1.00	–	<0.0001
	1. Minor problem	6,796	0.69	1.67	1.15, 2.43	
	2. Mild problem	9,508	1.09	2.52	1.88, 3.38	
	3. Moderately severe problem	10,626	1.28	2.75	2.07, 3.65	
	4. Severe to very severe problem	6,013	1.86	4.02	2.93, 5.51	
	Not done	33,856	0.61	1.56	1.22, 2.00	
4. Cognitive impairment	0. No problem	35,901	0.71	1.00	–	0.002
	1. Minor problem	12,139	0.96	1.24	0.95, 1.61	
	2. Mild problem	8,406	1.05	1.34	1.01, 1.77	
	3. Moderately severe problem	4,778	0.94	1.17	0.81, 1.70	
	4. Severe to very severe problem	1,263	0.87	1.03	0.48, 2.21	
	Not done	32,719	0.59	0.86	0.70, 1.05	
5. Physical impairment	0. No problem	39,112	0.92	1.00	–	<0.0001
	1. Minor problem	10,623	0.85	0.95	0.74, 1.22	
	2. Mild problem	7,714	0.56	0.70	0.51, 0.96	
	3. Moderately severe problem	3,959	0.48	0.59	0.38, 0.94	
	4. Severe to very severe problem	1,254	0.24	0.28	0.09, 0.91	
	Not done	32,544	0.59	0.71	0.58, 0.86	
6. Hallucinations/Delusions	0. No problem	22,967	0.72	1.00	–	0.02
	1. Minor problem	6,257	1.13	1.43	1.06, 1.93	
	2. Mild problem	10,790	0.90	1.03	0.76, 1.40	
	3. Moderately severe problem	13,347	0.76	0.96	0.72, 1.29	
	4. Severe to very severe problem	9,150	0.83	1.07	0.79, 1.46	
	Not done	32,695	0.60	0.83	0.66, 1.05	
7. Depressed mood	0. No problem	20,711	1.23	1.00	–	<0.0001
	1. Minor problem	12,167	0.84	0.71	0.54, 0.94	
	2. Mild problem	12,553	0.61	0.58	0.44, 0.76	
	3. Moderately severe problem	10,998	0.46	0.44	0.31, 0.61	
	4. Severe to very severe problem	6,061	0.49	0.50	0.34, 0.72	
	Not done	32,716	0.59	0.56	0.45, 0.69	
8. Other mental/Behavior problems	0. No problem	13,584	1.07	1.00	–	0.0003
	1. Minor problem	5,254	0.97	0.96	0.67, 1.37	
	2. Mild problem	16,946	0.62	0.60	0.45, 0.80	
	3. Moderately severe problem	18,009	0.70	0.70	0.54, 0.93	
	4. Severe to very severe problem	8,079	0.93	0.92	0.66, 1.27	
	Not done	33,334	0.61	0.64	0.50, 0.82	
9. Relationships	0. No problem	15,526	0.53	1.00	–	<0.0001
	1. Minor problem	13,259	0.60	1.08	0.79, 1.47	
	2. Mild problem	19,106	0.81	1.33	1.00, 1.78	
	3. Moderately severe problem	10,527	1.26	2.09	1.55, 2.82	
	4. Severe to very severe problem	3,848	1.72	2.62	1.80, 3.82	
	Not done	32,940	0.58	1.07	0.82, 1.39	
10. Daily living	0. No problem	28,931	0.75	1.00	–	0.006
	1. Minor problem	13,869	0.76	0.96	0.74, 1.25	
	2. Mild problem	12,037	1.03	1.31	1.02, 1.68	
	3. Moderately severe problem	6,201	0.81	1.10	0.79, 1.51	
	4. Severe to very severe problem	1,564	1.28	1.57	0.90, 2.72	
	Not done	32,604	0.58	0.82	0.66, 1.01	
11. Living conditions	0. No problem	38,859	0.52	1.00	–	<0.0001
	1. Minor problem	9,127	0.80	1.44	1.08, 1.92	
	2. Mild problem	6,157	1.28	2.03	1.51, 2.74	
	3. Moderately severe problem	3,444	1.77	2.76	1.96, 3.90	
	4. Severe to very severe problem	3,719	2.47	3.76	2.76, 5.13	
	Not done	33,900	0.60	1.13	0.92, 1.39	
12. Occupation/Activities	0. No problem	33,987	0.63	1.00	–	<0.0001
	1. Minor problem	10,516	0.82	1.25	0.96, 1.64	
	2. Mild problem	9,339	1.07	1.48	1.13, 1.94	
	3. Moderately severe problem	4,467	1.59	2.27	1.68, 3.08	
	4. Severe to very severe problem	2,440	1.72	2.31	1.55, 3.43	
	Not done	34,457	0.57	0.90	0.73, 1.11	
**HoNOS items at discharge**						
1. Aggression/Overactivity	0. No problem	41,595	0.44	1.00	–	<0.0001
	1. Minor problem	16,420	0.79	1.59	1.25, 2.03	
	2. Mild problem	4,069	2.19	4.15	3.11, 5.53	
	3. Moderately severe problem	1,033	4.65	8.62	5.87, 12.65	
	4. Severe to very severe problem	443	11.74	22.37	15.29, 32.73	
	Not done	31,646	0.65	1.43	1.16, 1.77	
2. Self-harm	0. No problem	52,106	0.79	1.00	–	0.27
	1. Minor problem	6,913	0.77	1.11	0.83, 1.47	
	2. Mild problem	2,711	0.66	0.97	0.60, 1.56	
	3. Moderately severe problem	1,190	1.09	1.56	0.88, 2.76	
	4. Severe to very severe problem	630	1.27	1.79	0.88, 3.66	
	Not done	31,656	0.65	0.88	0.73, 1.06	
3. Substance abuse	0. No problem	41,652	0.47	1.00	–	<0.0001
	1. Minor problem	8,339	0.79	1.45	1.05, 2.01	
	2. Mild problem	7,220	1.27	2.20	1.64, 2.96	
	3. Moderately severe problem	4,616	1.91	3.11	2.28, 4.23	
	4. Severe to very severe problem	1,471	3.87	6.71	4.68, 9.62	
	Not done	31,908	0.65	1.30	1.06, 1.59	
4. Cognitive impairment	0. No problem	45,917	0.71	1.00	–	0.11
	1. Minor problem	11,884	0.88	1.11	0.86, 1.43	
	2. Mild problem	4,193	1.19	1.50	1.05, 2.13	
	3. Moderately severe problem	1,205	1.41	1.56	0.82, 2.97	
	4. Severe to very severe problem	285	1.75	2.22	0.82, 5.99	
	Not done	31,722	0.65	0.93	0.77, 1.12	
5. Physical impairment	0. No problem	45,648	0.89	1.00	–	0.0004
	1. Minor problem	9,881	0.55	0.65	0.48, 0.88	
	2. Mild problem	5,259	0.63	0.82	0.57, 1.17	
	3. Moderately severe problem	2,109	0.47	0.62	0.34, 1.13	
	4. Severe to very severe problem	622	0.16	0.22	0.04, 1.36	
	Not done	31,687	0.65	0.78	0.65, 0.94	
6. Hallucinations/Delusions	0. No problem	38,935	0.78	1.00	–	0.002
	1. Minor problem	12,306	0.72	0.87	0.66, 1.15	
	2. Mild problem	9,609	0.66	0.85	0.63, 1.15	
	3. Moderately severe problem	2,051	1.51	2.09	1.44, 3.03	
	4. Severe to very severe problem	571	2.28	2.69	1.39, 5.23	
	Not done	31,734	0.66	0.87	0.72, 1.06	
7. Depressed mood	0. No problem	34,440	0.98	1.00	–	0.0003
	1. Minor problem	18,006	0.62	0.71	0.57, 0.88	
	2. Mild problem	8,488	0.47	0.57	0.41, 0.78	
	3. Moderately severe problem	2,075	0.53	0.67	0.39, 1.15	
	4. Severe to very severe problem	511	0.39	0.45	0.12, 1.75	
	Not done	31,686	0.65	0.72	0.59, 0.88	
8. Other mental/Behavior problems	0. No problem	32,356	0.80	1.00	–	<0.0001
	1. Minor problem	12,714	0.50	0.67	0.50, 0.90	
	2. Mild problem	13,240	0.74	1.01	0.78, 1.31	
	3. Moderately severe problem	3,726	1.45	1.89	1.36, 2.61	
	4. Severe to very severe problem	840	2.26	3.07	1.86, 5.06	
	Not done	32,330	0.66	0.88	0.72, 1.08	
9. Relationships	0. No problem	26,650	0.44	1.00	–	<0.0001
	1. Minor problem	18,075	0.55	1.15	0.88, 1.52	
	2. Mild problem	13,873	1.19	2.40	1.85, 3.12	
	3. Moderately severe problem	3,812	2.12	4.06	2.92, 5.63	
	4. Severe to very severe problem	908	4.41	9.03	6.07, 13.45	
	Not done	31,888	0.64	1.40	1.10, 1.79	
10. Daily living	0. No problem	44,752	0.72	1.00	–	0.02
	1. Minor problem	11,721	0.83	1.11	0.87, 1.42	
	2. Mild problem	5,381	1.28	1.62	1.22, 2.17	
	3. Moderately severe problem	1,319	1.06	1.35	0.68, 2.66	
	4. Severe to very severe problem	324	1.23	1.96	0.79, 4.88	
	Not done	31,709	0.64	0.93	0.77, 1.12	
11. Living conditions	0. No problem	47,609	0.55	1.00	–	<0.0001
	1. Minor problem	8,954	0.93	1.48	1.12, 1.94	
	2. Mild problem	3,754	1.31	2.05	1.42, 2.95	
	3. Moderately severe problem	1,260	2.46	3.49	2.11, 5.79	
	4. Severe to very severe problem	1,067	6.09	9.07	6.34, 12.97	
	Not done	32,562	0.67	1.21	1.00, 1.47	
12. Occupation/Activities	0. No problem	42,725	0.63	1.00	–	<0.0001
	1. Minor problem	11,462	0.81	1.19	0.91, 1.55	
	2. Mild problem	5,979	1.25	1.79	1.33, 2.39	
	3. Moderately severe problem	1,541	2.01	2.60	1.62, 4.18	
	4. Severe to very severe problem	704	3.27	4.81	2.98,7.74	
	Not done	32,795	0.66	1.07	0.88, 1.30	

The predominant diagnosis of the “other mental health diagnoses” category was personality disorder (F6), which increased over time, and was associated with follow-up in prison. This trend therefore explained some of the increased number of prison follow-ups, and is discussed further below.

In considering model of care variables, bivariate analyses showed significant differences by region (*p* = 0.0005) and number of seclusion events (*p* < 0.0001). Seclusion events during the inpatient admission also elevated the OR for prison referral following discharge (1 seclusion event OR = 3.51, 2+ seclusion events OR = 4.51; *p* < 0.0001) (see Table 2). Shorter lengths of stay were statistically more likely to result in subsequent imprisonment, with admissions of 0–6 days attracting more than twice the odds imprisonment when compared to admission durations of 13–24 days (OR = 2.38; *p* < 0.0001) (see Table 2). Individuals discharged on a Mental Health Act order had significantly increased odds of subsequent prison referral (OR = 1.7, *P* < 0.0001) (see Table 2).

### HoNOS

Health of the Nation Outcome Scale scores at admission and discharge provided an objective record of symptom severity and social information. In general, discharge ratings were more powerful predictors of prison referral to mental health than admission ratings (see Table 3).

#### Behavior subscale (aggression, self-harm, and substance abuse)

Those with severe to very severe aggression/overactivity (HoNOS item 1) either on admission or on discharge from hospital were at much higher risk of subsequent prison referral (OR = 3.57 at admission, 22.37 at discharge; *p* < 0.0001). The data did not find a significant relationship between self-harm (HoNOS item 2) at discharge and subsequent prison referral.

#### Impairment subscale (cognitive impairment and physical impairment)

Cognitive impairment problems (item 4) at discharge were not correlated with prison referral, whereas physical impairment problems (item 5) at discharge were negatively correlated, suggesting they operate as a protective factor.

#### Symptom subscale (hallucinations/delusions, depressed mood, and other mental/behavioral problems)

All three symptom clusters at discharge were significantly more likely to result in post-discharge imprisonment. The “severe to very severe” category increased the risk of prison referral by odds of 2.69 for delusions/hallucinations (*p* = 0.002), while “severe to very severe” behavioral problems increased the odds of imprisonment by 3.07 (*p* < 0.0001). Depression at discharge significantly reduced the odds of post-discharge imprisonment (*p* = 0.0003).

#### Social subscale (relationships, daily living, living conditions, and occupation/activities)

The HoNOS also considers a range of social variables including relationships, daily living, living conditions, and occupation/activities (items 9–12). Relationships (item 9), living conditions (item 11), and occupation/activities (item 12) were all significant predictors of post-discharge imprisonment (*p* < 0.0001), while daily living (item 10) was also significant (*p* = 0.02). The “severe to very severe” category at discharge increased the odds of post-discharge prison referral by odds of 9.03 (for item 9), 1.96 (for item 10), 9.07 (for item 11), and 4.81 (for item 12).

### Multiple variable results

The final multiple variable model is presented in Table 4. It includes the best subset of demographic and clinical measures and items of the HoNOS clinical scale at admission and at discharge.

**TABLE 4:** Final multiple variable model for custodial remand within 28 days.

		Adjusted OR	95% CI	Significant risk or protective factors	*p*-value
**Socio-demographics variables**					
Gender	Female	1.00	–		<0.0001
	Male	3.97	3.09, 5.09	R	
Ethnicity	Mâori	1.94	1.59, 2.37	R	<0.0001
	Pacific	2.04	1.44, 2.90	R	
	Other	1.39	0.92, 2.12		
	NZ European/Pakeha	1.00	–		
Age	16–20	1.88	1.24, 2.87	R	<0.0001
	20–25	2.70	1.84, 3.98	R	
	26–30	2.65	1.79, 3.92	R	
	31–40	2.73	1.88, 3.97	R	
	41–50	1.77	1.17, 2.66	R	
	51+	1.00	–		
**Clinical characteristics**					
Year discharged	2012	1.00	–		0.26
	2013	0.98	0.65, 1.48		
	2014	1.19	0.78, 1.82		
	2015	1.43	0.95, 2.13		
	2016	1.44	0.96, 2.16		
	2017	1.43	0.97, 2.11		
	2018	1.19	0.80, 1.76		
	2019	1.33	0.91, 1.96		
	2020	1.08	0.73, 1.60		
Primary diagnosis (ICD 10 codes)	Psychoactive substance use (F1)	2.00	1.47, 2.72	R	<0.0001
	Psychotic disorders (F2)	1.30	1.01, 1.68	R	
	Mood disorders (F3)	0.83	0.60, 1.14		
	Anxiety and other non-psychotic mental disorders (F4)	1.42	0.94, 2.16		
	Other mental health diagnoses (F)	1.81	1.15, 2.84	R	
	Other diagnoses	1.40	0.92, 2.11		
	Not diagnosed	1.00	–		
**Model of care variables**					
Region	Auckland RFPS	1.00	–		0.0008
	Canterbury RFPS	0.93	0.69, 1.26		
	Central RFPS	0.93	0.69, 1.25		
	Midland RFPS	1.37	1.05, 1.80	R	
	Southern RFPS	1.65	1.17, 2.34	R	
Seclusion events	0	1.00	–		<0.0001
	1	2.33	1.84, 2.97	R	
	2+	3.60	2.67, 4.86	R	
Length of stay (days)	0–6	1.00	–		<0.0001
	7–12	0.74	0.60, 0.91	P	
	13–24	0.62	0.49, 0.79	P	
	25+	0.43	0.32, 0.57	P	
**HoNOS items at admission**					
3. Substance abuse	0. No problem	1.00	–		0.03
	1. Minor problem	1.17	0.81, 1.69		
	2. Mild problem	1.42	1.06, 1.91	R	
	3. Moderately severe problem	1.38	1.04, 1.82	R	
	4. Severe/Very severe problem	1.72	1.24, 2.37	R	
	Not done	1.30	0.73, 2.34		
6. Hallucinations/Delusions	0. No problem	1.00	–		0.007
	1. Minor problem	1.18	0.86, 1.61		
	2. Mild problem	0.81	0.59, 1.12		
	3. Moderately severe problem	0.68	0.49, 0.93	P	
	4. Severe/Very severe problem	0.63	0.45, 0.90	P	
	Not done	1.26	0.51, 3.11		
7. Depressed mood	0. No problem	1.00	–		0.007
	1. Minor problem	0.78	0.59, 1.02		
	2. Mild problem	0.68	0.51, 0.90	P	
	3. Moderately severe problem	0.54	0.38, 0.78	P	
	4. Severe/Very severe problem	0.65	0.42, 1.02		
	Not done	1.13	0.45, 2.81		
8. Other mental/Behavior problems	0. No problem	1.00	–		0.03
	1. Minor problem	1.17	0.82, 1.67		
	2. Mild problem	0.71	0.54, 0.94	P	
	3. Moderately severe problem	0.82	0.62, 1.07		
	4. Severe/Very severe problem	0.93	0.65, 1.32		
	Not done	1.33	0.73, 2.40		
11. Living conditions	0. No problem	1.00			0.004
	1. Minor problem	1.26	0.92, 1.73		
	2. Mild problem	1.55	1.14, 2.12	R	
	3. Moderately severe problem	1.65	1.13, 2.41	R	
	4. Severe to very severe problem	2.05	1.43, 2.93	R	
	Not done	1.35	0.73, 2.50		
12. Occupation/Activities	0. No problem	1.00	–		0.0004
	1. Minor problem	1.10	0.82, 1.47		
	2. Mild problem	1.04	0.78, 1.39		
	3. Moderately severe problem	1.49	1.06, 2.10	R	
	4. Severe/Very severe problem	1.09	0.71, 1.67		
	Not done	0.30	0.13, 0.71	P	
**HoNOS items at discharge**					
1. Aggression/Overactivity	0. No problem	1.00	–		<0.0001
	1. Minor problem	1.36	1.06, 1.74	R	
	2. Mild problem	2.66	1.95, 3.62	R	
	3. Moderately severe problem	3.85	2.51, 5.90	R	
	4. Severe/Very severe problem	8.96	5.59, 14.38	R	
	Not done	7.51	1.30, 43.35	R	
5. Physical impairment	0. No problem	1.00	–		<0.0001
	1. Minor problem	0.70	0.51, 0.95	P	
	2. Mild problem	0.78	0.52, 1.17		
	3. Moderately severe problem	0.56	0.30, 1.06		
	4. Severe/Very severe problem	0.08	0.01, 0.72	P	
	Not done	2.35	0.28, 19.82		
6. Hallucinations/Delusions	0. No problem	1.00	–		0.03
	1. Minor problem	0.76	0.58, 1.00		
	2. Mild problem	0.64	0.46, 0.88	P	
	3. Moderately severe problem	0.87	0.57, 1.33		
	4. Severe/Very severe problem	0.76	0.39, 1.51		
	Not done	2.50	0.85, 7.35		
9. Relationships	0. No problem	1.00	–		<0.0001
	1. Minor problem	1.08	0.83, 1.42		
	2. Mild problem	1.64	1.24, 2.19	R	
	3. Moderately severe problem	1.86	1.28, 2.69	R	
	4. Severe/Very severe problem	2.58	1.56, 4.25	R	
	Not done	0.02	0.00, 0.39	P	
11. Living conditions	0. No problem	1.00	–		0.006
	1. Minor problem	1.08	0.82, 1.43		
	2. Mild problem	0.98	0.68, 1.41		
	3. Moderately severe problem	1.46	0.90, 2.36		
	4. Severe/Very severe problem	2.41	1.56, 3.73	R	
	Not done	1.95	1.08, 3.51	R	

It was recognized that due to the COVID-19 pandemic, 2020 may have experienced different service use patterns than other years. However, the exclusion of 2020 did not change the results, therefore it was left in the analysis.

The significant demographic variables included in the final multiple variable model demonstrated higher odds for men, higher odds for Mâori in comparison to European; and higher odds for younger age groups.

The significant clinical variables included specific diagnostic categories: ICD 10 diagnostic codes related to psychoactive substance use, and psychotic disorders.

Some HoNOS items correlated negatively with post-discharge imprisonment, including depression/mood problems at admission, physical impairment problems at discharge, and hallucinations/delusions problems at either admission or discharge. The significant risk factors were self-harm at admission, substance abuse problems at admission, other mental health problems at admission, aggression/overactivity at discharge, relationship problems at discharge, and living conditions at admission and discharge.

The significant model of care variables included the region in which the inpatient admission occurred (New Zealand is divided into five geographic regions in the analysis), the increased risk associated with a greater number of seclusion events during the preceding hospitalization and higher odds for shorter lengths of stay during hospitalization (see Table 4).

The overall conclusion is that the observed significant increase in reception to a remand prison within 28 days of acute mental health services discharge is not a uniform increase. It can be explained by an increasing proportion of at risk patients discharged over time being: demographically younger, male, and Mâori; clinically suffering more commonly with psychotic disorders and substance use disorders, experiencing a shorter admission with more seclusion while in hospital and being more aggressive/overactive at discharge from hospital; and socially experiencing living condition and relationship problems at discharge.

## Discussion

The research question was raised because of concern that changing models of care in adult mental health services might be leading to a progressive transfer of clinical responsibility to the criminal justice system, a concern increasingly raised in other jurisdictions (21). We hypothesized that any transfer of care to prison based mental health teams would be more likely to affect people presenting with disturbed behavior, which has in the past been managed with coercive practices no longer supported by contemporary models of care, in an environment of reducing mental health bed availability. We were also concerned that although prisons are resourced to manage aggressive and violent behavior, they do so without the clinical environment, resources or staff necessary to appropriately treat people suffering from serious mental disorders. In addition, compulsory treatment is not permitted in New Zealand prison settings. Therefore, if such a trend is emerging, the human rights of affected persons would appear to be undermined rather than supported by the aforementioned policy shifts.

Over the 9-year study period that we examined, there were increasing numbers of individuals who, after discharge from mental health services, were followed up in prison. Over the same time period mental health discharges slightly decreased (see Table 1). Consequently, the rate of imprisonment following discharge from mental health units is shown to have significantly increased. However, once we adjusted for the at-risk characteristics, this time trend was no longer statistically significant. Therefore, the observed increase in imprisonment rate could be explained by the at-risk characteristics of the population changing over time, including changes in demographic, clinical, social, and “model of care” variables. In other words, it appeared that people discharged from inpatient units in Aotearoa increasingly have the characteristics of people who have always been at higher risk of imprisonment (younger men of Mâori and Pacific ethnicity with psychotic and drug related clinical conditions who are subject to seclusion during relatively short admissions).

For reasons which were unclear, a personality disorder diagnosis at discharge also increased over the study period, and was also associated with imprisonment following discharge. With fewer inpatient beds and shorter lengths of stay, it is difficult to understand the clinical justification for this trend, although it may relate to the dearth of alternative residential options for personality disordered patients presenting in crisis.

Imprisonment following inpatient unit discharge is now an outcome facing nearly 1% of all adult inpatient discharges.

Although the demographic characteristics of the group at high risk of imprisonment following inpatient care are also disproportionately shared by the general prison population (16), a focus on equity would see greater resources directed to this population during inpatient admissions and at the point of inpatient discharge. Readmission to an acute psychiatric unit within 28 days of discharge is a commonly monitored key performance indicator (KPI) for adult mental health services (22). We suggest remand to prison within 28 days of discharge from an acute psychiatric unit may also implicate incomplete or ineffective treatment, and could also be monitored as a KPI.

The increased recreational use of methamphetamine and synthetic cannabinoid analogs in the community over the study period has been dramatic, as have the mental health sequelae (23), including substance induced psychotic disorders requiring an inpatient admission. We believe the observed increase in risk of imprisonment following discharge associated with aggression (both on admission and discharge) over the study period is likely to be related to the clinical diagnosis changes observed. A greater clinical focus on the post-discharge care of people with these disorders may be indicated.

The increasing level of homelessness and social deprivation in the New Zealand community over the time of this study has also been well publicized (24), making well supported community placements very challenging to secure at discharge, particularly when inpatient occupancy levels are regularly above 100% and precipitous discharges are necessary to make room for the next admission (25). There is an urgent need for more social resources for people recently discharged from inpatient psychiatric care.

Regarding seclusion, the Ministry of Health reported a 13% reduction in the number of people who experienced seclusion during an inpatient admission from 2009 to 2020 (26). Given this study’s findings, it appears those still experiencing seclusion are now at higher risk of imprisonment shortly following discharge. Although there are a range of efforts to implement alternatives to seclusion (13), we suggest new models of care must be able to effectively manage aggressive and sometimes violent behavior known to be signs of some clinical conditions (27). The present paper suggests that contemporary models of care in inpatient mental health units may not yet provide satisfactory management solutions for some admitted people, even though alternative strategies have been reported to have a “*reasonably high degree of evidence for effectively reducing the use of coercive measures in clinical practice*” (9).

In terms of other coercive interventions, national data showed gradual increasing use of the Mental Health Act over the period of study (26). In our analysis, legal status was included in the multivariate model, but when HoNOS variables were included, it failed to add to the power of the regression model. We concluded that a combination of HoNOS items (including “aggression/overactivity” and “symptoms” which are part of the legal test for mental disorder) were independently capturing this risk.

In terms of social factors, this study suggests that people discharged from inpatient mental health units in New Zealand are increasingly struggling to secure stable and supportive accommodation, and meaningful employment, while their relationships are under more stress. Perhaps we should not be surprised that they are increasingly being remanded into prison following discharge.

Ministry of Health data revealed that regional differences in mental health bed numbers did not explain the regional differences in outcomes, although they have fallen well below international benchmark standards in all regions. Unless clinical presentations fall, fewer beds will drive shorter LOS, which we have shown is correlated with imprisonment within 28 days of inpatient discharge. The current level of investment in community mental health care appears insufficient to manage people returning to the community after these very brief admissions, in line with earlier research findings (3). The idea that as the number of psychiatric hospital beds falls, more prison beds are needed was first suggested by Penrose (28). Although a recent systematic review of cohort studies did not find general support for this hypothesis in deinstitutionalised long-stay populations (21), the authors queried whether new populations may be impacted negatively if they cannot access psychiatric hospital care. It may be that with very low mental health bed numbers and less coercive models of care, a type of “Penrose effect” is now emerging in response to more recent human rights and clinical environment developments. In the UK, serious concerns have been voiced regarding the consequences of failing mental health systems for the criminal justice sector (29).

## Limitations

This study was limited by information available in the national mental health dataset, which did not include criminal records. Although we have reported and analysed national HoNOS data, this was missing in a third of cases which required accommodation in the statistical analysis. Further, ratings were made by unblinded treating clinicians potentially exposing rating biases. While clinicians are provided training in administering the HoNOS ratings, data quality may have suffered from the pressure of clinical demands.

## Conclusion

People who were discharged from acute mental health units on a Mental Health Act order, after a short admission during which they were secluded, and who presented with behavioral symptoms related to psychosis and drug use were at higher risk of imprisonment in the post-discharge period. Affected people also showed a trend toward being younger, of Mâori or Pacific descent, with compromised social supports, and appeared to be poorly served by contemporary models of care. Greater resources need to be applied to these cases to reduce the risk of imprisonment following inpatient discharge. This includes sufficient beds to avoid early discharge into unsafe community care.

We further believe more effort is needed to replace coercive practices with effective alternatives, which do not see behavior driven by mental illness as a criminal justice issue. Such alternatives will need to be co-designed with key stakeholders (including those with lived experience of such practices) and evidence for their effectiveness determined. Until evidence for this is more robustly available, it may be premature to abandon completely the use of some capacity for coercion in inpatient mental health units. Mental health services must continue to embrace all behavior driven by mental illness as clinical issues, for which clinical services can provide safe and appropriate care. To recast these behaviors as a criminal issue would, in our view, abandon our clinical and ethical responsibilities to the population identified by this study.

## Data availability statement

The data analyzed in this study is subject to the following licenses/restrictions: the dataset is managed and accessed through the New Zealand Ministry of Health. Requests to access these datasets should be directed to Data-enquiries@health.govt.nz.

## Ethics statement

The studies involving human participants were reviewed and approved by the Ministry of Health’s Health and Disability Ethics Committee. A letter of approval was obtained as a result of an expedited research application. Written informed consent for participation was not required for this study in accordance with the national legislation and the institutional requirements.

## Author contributions

All authors contributed to the study concept, design, analysis, interpretation, reviewed, and approved the final version of the manuscript.

## Footnotes

1 Personal communication.

2
www.sas.com
